# Segmentally Demineralized Cortical Bone With Stem Cell-Derived Matrix Promotes Proliferation, Migration and Differentiation of Stem Cells *in vitro*

**DOI:** 10.3389/fcell.2021.776884

**Published:** 2022-01-26

**Authors:** Shu-Kun He, Liang-Ju Ning, Ruo-Nan Hu, Xuan Yao, Jing Cui, Wei Ding, Jing-Cong Luo, Ting-Wu Qin

**Affiliations:** ^1^ Laboratory of Stem Cell and Tissue Engineering, State Key Laboratory of Biotherapy, West China Hospital, Orthopedic Research Institute, Sichuan University, Chengdu, China; ^2^ Department of Orthopedics, West China Hospital, Orthopedic Research Institute, Sichuan University, Chengdu, China; ^3^ Department of Orthopedics, First Affiliated Hospital, College of Medicine, Zhejiang University, Hangzhou, China; ^4^ Department of Clinical Hematology, Faculty of Laboratory Medicine, Army Medical University, Chongqing, China

**Keywords:** segmentally demineralized cortical bone, stem cell-derived matrix, proliferation, migration, differentiation

## Abstract

A recent study has shown that demineralized cortical bone (DCB) did not improve the healing of tendon-bone interface. Considering that there is a gradient of mineral content in the tendon-bone interface, we designed a segmentally demineralized cortical bone (sDCB) scaffold with two different regions: undemineralized cortical bone section within the scaffold (sDCB-B) and complete demineralized cortical bone section within the scaffold (sDCB-D), to mimic the natural structure of the tendon-bone interface. Furthermore, the extracellular matrix (ECM) from tendon-derived stem cells (TDSCs) was used to modify the sDCB-D region of sDCB to construct a novel scaffold (sDCB-ECM) for enhancing the bioactivity of the sDCB-D. The surface topography, elemental distribution, histological structure, and surface elastic modulus of the scaffold were observed using scanning electron microscopy, energy-dispersive X-ray spectroscopy, Fourier transform infrared spectroscopy, histological staining and atomic force microscopy. Cell proliferation of bone marrow mesenchymal stem cells (BMSCs) and TDSCs cultured on scaffolds was evaluated using the Cell Counting kit-8, and cell viability was assessed by Live/Dead cell staining. Cell morphology was detected by fluorescent staining. The ability of the scaffolds to recruit stem cells was tested using transwell migration assay. The expression levels of bone-, cartilage- and tendon-related genes and proteins in stem cells were assessed by the polymerase chain reaction and western blotting. Our results demonstrated that there was a gradient of Ca and P elements in sDCB, and TDSC-derived ECM existed on the surface of the sDCB-D region of sDCB. The sDCB-ECM could promote stem cell proliferation and migration. Moreover, the sDCB-B region of sDCB-ECM could stimulate osteogenic and chondrogenic differentiation of BMSCs, and the sDCB-D-ECM region of sDCB-ECM could stimulate chondrogenic and tenogenic differentiation of TDSCs when compared to DCB. Our study indicated that sDCB-ECM might be a potential bioscaffold to enhance the tendon-bone interface regeneration.

## Introduction

The tendon-bone interface is difficult to repair because of its highly heterogeneous and complex structure with gradually increasing mineral content (Isaac et al., 2012). This unique area consists of four different regions: tendon, fibrocartilage, mineralized fibrocartilage and bone (Genin et al., 2009). The demineralized bone matrix (DBM) scaffold consists of the type I collagen and several growth factors, such as transforming growth factor-*β*1 (TGF-*β*1) and insulin-like growth factors-1 (Blum et al., 2004). Several research groups have used DBM to repair the tendon-bone interface. Jackson et al. (1996) found that a fibrocartilage transition area could be seen when DBM was used to reconstruct the anterior cruciate ligament in goats. Sundar et al. (2009) also found that DBM could improve the healing of the tendon-bone interface of the ovine patellar tendon, and the DBM group showed better functional weight-bearing than the control group. Elnikety et al. (2016) discovered that demineralized cortical bone (DCB) was remodeled into the neo-enthesis when using DCB to repair ovine patellar tendon defects. However, a recent study reported that DBM did not improve the healing of the tendon-bone interface in a rat rotator cuff tear model (Thangarajah et al., 2017).

Considering the structure of the natural tendon-bone interface, other studies have developed a continuous hard-soft scaffold from cancellous bones (Dickerson et al., 2013; Boys et al., 2019). Their results showed that regionally demineralized cancellous bone accelerated the healing of the fibrocartilaginous interface in an ovine rotator cuff tear model. Inspired by the heterogeneous structure of the natural tendon-bone interface, we attempted to prepare a segmentally demineralized cortical bone (sDCB) scaffold with two different regions: undemineralized bone section within the scaffold (sDCB-B) and complete demineralized bone section within the scaffold (sDCB-D), to mimic the natural structure of the tendon-bone interface. Our previous studies demonstrated that DCB treatment with H_2_O_2_ could reduce the risk of heterotopic ossification and might be used for tendon repair (Yang et al., 2018a; Qing et al., 2019). However, H_2_O_2_-treated DCB lacked biochemical components that can induce stem cell migration and differentiation.

Cell-based therapy is a promising approach for regenerative medicine. Several authors have found that a combination of DBM and bone marrow mesenchymal stem cells (BMSCs) could promote the healing of the enthesis in ovine and rat models (Thangarajah et al., 2016; Thangarajah et al., 2018). Although exogenous stem cells can repair tissue injuries, the method is time-consuming, involving costly cell culture steps, and has a low survival rate of cells *in vivo* (Ikada, 2006; Bernardo et al., 2011). Therefore, an *in-situ* strategy, facilitating tissue regeneration by recruiting endogenous stem cells, has been developed (Shen et al., 2010; Tang et al., 2016; Pacelli et al., 2017). The extracellular matrix (ECM) is a complex structure with bioactive compounds, that can regulate cell proliferation, migration and differentiation (Reilly and Engler, 2010). The ECM can be divided into tissue-derived ECM and cell-derived ECM. Previous studies have demonstrated that cell-derived ECM could provide a special microenvironment to accelerate the healing of injured tissues (Zhang et al., 2016). Some studies have developed scaffolds coated with cell-derived ECM, and these scaffolds could induce osteogenic differentiation of stem cells and promote bone regeneration in animal studies (Kim et al., 2015; Harvestine et al., 2016). For the tendon-bone interface, a recent study showed that tendons contained more stem cells than bones, and tendon-derived stem cells (TDSCs) might play a key role in enthesis regeneration (Campbell et al., 2019). In addition, TDSCs showed higher proliferative capacity and better differentiation potential than BMSCs (Tan et al., 2012). Thus, scaffolds coated with TDSC-derived ECM used for the tendon-bone interface are of concern and need to be investigated. In this study, we used the TDSC-derived ECM to modify the sDCB-D region of sDCB for enhancing the bioactivity of the sDCB-D. We hypothesized that the sDCB coated with the TDSC-derived ECM (sDCB-ECM) could enhance osteogenic differentiation of BMSCs at the sDCB-B region and tenogenic differentiation of TDSCs at the sDCB-D with ECM (sDCB-D-ECM) region when compared with DCB.

## Materials and Methods

### Preparation and Characterization of sDCB

All experiments were performed in accordance with the standard guidelines approved by the Sichuan University Animal Care and Use Committee (No. 2019155A). A combination of physical, chemical and enzymatic treatments was used to prepare the sDCB scaffold according to our published protocols with some modifications (Yang et al., 2018a; Qing et al., 2019). First, all soft tissues, including blood and marrow, were removed from fresh bovine bones (New Hope Group, China). Then, cortical bone cylinders (approximately 40 mm in length and 30 mm in diameter) were made using an automatic cutting machine (Q-80Z, Sanfeng Instrument Technology, Changzhou, China) under cool water condition. Sandpapers and files removed all cancellous bone in the cortical bone cylinders. Next, bone cylinders were manufactured into bone slices (10 mm × 5 mm × 0.5 mm) using an automated diamond wire saw (STX-202A, Kejing Auto-Instrument, Liaoning, China) along the long axis. Each bone slice was manually divided into two regions: undemineralized bone section within the bone slice (sDCB-D) and complete demineralized bone section within the bone slice (sDCB-D).

The sDCB-D region of sDCB was immersed in 0.6 N hydrochloric acid (HCl, Chron Chemicals, Chengdu, China) for 140 min at 25°C for complete demineralization according to previous studies (Lewandrowski et al., 1996; Summitt and Reisinger, 2003). After demineralization, samples were rinsed overnight in phosphate-buffered saline (PBS, HyClone, Logan, UT, United States). Next, the samples were degreased with methanol (Chron Chemicals) and ethanol (Chron Chemicals) (v/v, 1:1) for 12 h. After being rinsed in PBS, samples were decellularized with 0.5% Triton X-100 (Amresco, OH, United States), DNase (150 IU/ml, Roche, Mannheim, Germany), and RNase (100 μg/ml, Roche, Mannheim, Germany) for 24 h. Next, the sDCB-D region of sDCB was soaked in 3% hydrogen peroxide for 12 h to remove the inherent osteoinductivity. Finally, the sDCB was rinsed, lyophilized, and sterilized. The surface morphology and distribution of calcium (Ca) and phosphorus (P) in sDCB were characterized by field scanning electron microscopy (SEM, JSM-7500F, JEOL, Japan) with energy dispersive X-ray (EDX) detector. In addition, the composition within the two different regions (sDCB-B and sDCB-D) of sDCB was determined using Fourier transform infrared spectroscopy (FT-IR, Nicolet 6,700, Thermo Scientific, Waltham, MA, United States).

### Preparation and Characterization of sDCB-ECM

TDSCs and BMSCs from Sprague−Dawley (SD) rats were isolated and cultured according to our published protocol (Ning et al., 2015). In brief, TDSCs were isolated from Achilles tendons and flexor tendons of rats, and BMSCs were isolated from the bone marrow of femur and tibia. Then, TDSCs and BMSCs were cultured in a culture medium (low-glucose Dulbecco’s modified Eagle’s medium (L-DMEM), 20% fetal bovine serum (FBS) and 1% penicillin/streptomycin (Gibco, Amarrilo, TX, United States). The culture medium was changed every 3 days, and all experiments were performed with TDSCs and BMSCs at passage 3.

TDSCs were seeded on the surface of the sDCB-D region of sDCB at a density of 5×10^4^ cells per scaffold. Cells were cultured in a culture medium that was changed every 2 days. On the sixth day, 50 μM ascorbic acid (Sigma, St Louis, MO, United States) was added to the subsequent medium to promote the deposition of TDSC-derived ECM. After 2 weeks, the sDCB-D coated with TDSCs was decellularized with 0.5% Triton X-100. Furthermore, we set four different time points: 2, 5, 10, and 15 min to obtain the optimal decellularization time. The deoxyribonucleic acid (DNA) of each sample (*n* = 3 per group) was extracted using a tissue DNA isolation kit (Omega Biotek, GA, United States) and quantified using NanoDrop spectrophotometer (Thermo Fisher, United States). After decellularization, the sDCB-D-ECM region was formed, and the whole scaffold was named sDCB-ECM including two different regions: sDCB-B and sDCB-D-ECM. The surface morphologies of sDCB-D, sDCB-D with TDSCs and sDCB-D-ECM were characterized using the SEM (EVO 10, Carl Zeiss, Jena, Germany). In addition, the same samples from each group were fixed in 4% paraformaldehyde, embedded in paraffin, and longitudinally cut into 5 μm thick sections. Hematoxylin and eosin (H&E) and Masson’s trichrome staining were used to observe the cellular components and ECM of scaffolds. The biochemical compositions of sDCB and sDCB-ECM, including the stromal cell-derived factor 1 (SDF-1), biglycan (Bgn) and fibromodulin (Fmod), were quantified by the enzyme-linked immunosorbent assay (ELISA, SDF-1, DLDEVELOP, China; Bgn, Cloud-Clone, United States; Fmod, DLDEVELOP, China, *n* = 3 per group). Finally, the surface elastic modulus of sDCB-B, sDCB-D and sDCB-D-ECM was determined by an atomic force microscope (AFM, Cypher VRS, Asylum Research, MA, United States).

### Cell Attachment, Viability and Proliferation Assay

We evaluated the effect of each region of the scaffold separately on the stem cell attachment, viability, and proliferative capacity since the sDCB-ECM contained two different regions. Specifically, we used BMSCs to evaluate the effect of sDCB-B, and TDSCs to evaluate the effect of sDCB-D-ECM, based on the different regions where the scaffolds promoted regeneration. DCB was used as the control group. Briefly, DCB, sDCB-B and sDCB-D-ECM were placed in 24-well plates (Corning, NY, United States). BMSCs were seeded on the DCB and sDCB-B at a density of 5 × 10^3^ cells/cm^2^ (*n* = 3 per group), and TDSCs were seeded on the DCB and sDCB-D-ECM at a density of 5 × 10^3^ cells/cm^2^ (*n* = 3 per group) in culture medium. At 24 h after culture, to assess the effect of each region of the scaffold on cell attachment, first, the cell-seeded scaffolds were fixed in 4% paraformaldehyde and pretreated with 0.5% Triton X-100. Then, rhodamine-phalloidin (Abcam, United States) and 4,6-diamidino-2-phenylindole (DAPI, Sigma, United States) were used to stain the cytoskeleton and nucleus, respectively. Finally, the cells on the scaffolds were observed using a fluorescent microscope (Axio Imager Z2, Zeiss, Jena, Germany). Meanwhile, a Live/Dead assay kit (Beyotime, Shanghai, China) including calcein-AM and propidium iodide (PI) was used to assess the effect of each scaffold region on cell viability. The cell-seeded scaffolds were incubated for 30 min in a culture medium supplemented with 2 μM calcein-AM and 4 μM PI, and the scaffolds were observed by fluorescence microscopy (Axio Imager Z2, Zeiss, Jena, Germany). At 1, 3, 5, 7 days after culture, the proliferation of cells on the scaffolds was quantified using the Cell Counting kit-8 (CCK-8, Dojindo, Tabaru, Japan). The cells-seeded scaffolds were incubated in fresh medium without FBS, and then 10% CCK-8 solution was added to each well. After incubation at 37°C for 4 h, the scaffolds were removed and the absorbance of medium was measured at 450 nm using a spectrophotometer (Synergy H1, Bio-Tek, VT, United States).

### Transwell Migration Assay

The recruitment capacity of sDCB-ECM was evaluated using a 24-well chamber transwell chemotactic migration model (pore size: 8 μm, Corning, United States, *n* = 3 per group). The DCB was used as the control group, and two different regions of the sDCB-ECM were evaluated separately. First, DCB, sDCB-B or sDCB-D-ECM was put in the lower chamber, and 200 μL of BMSCs or TDSCs suspension (5 × 10^5^ cells/ml) was placed in the upper chamber after serum-starving for 12 h. Next, 1 ml L-DMEM supplemented with 1% FBS was added to the lower chamber. After incubation at 37°C for 12 h, non-migrated cells in the upper chamber were scraped with cotton swabs, and the cells that migrated through the pores were fixed with 4% paraformaldehyde. After staining with DAPI, BMSCs or TDSCs on the lower surface of the membrane were captured by a fluorescence microscope (Axio Imager Z2, Zeiss, Jena, Germany). Three random fields were obtained at ×100 magnification to calculate the mean number of cells in each well using ImageJ software (NIH, Bethesda, MA, United States).

### Polymerase Chain Reaction (PCR)

At 7 and 14 days after culture, total RNA (*n* = 3 per group) was extracted by lysing the cells using the TRIZOL reagent (Invitrogen, Waltham, MA, United States) following the manufacturer’s protocol. The cDNA was synthesized from total RNA by reverse transcription using the GoScript Reverse Transcription System (Promega, Madison, WI, United States). The rat-specific primers for genes, including glyceraldehyde-3-phosphate dehydrogenase (*GAPDH*), collagen I (*COL1*), runt-related transcription factor 2 (*RUNX2*), alkaline phosphatase (*ALP*), osteocalcin (*OCN*), osteopontin (*OPN*), SRY-box transcription factor 9 (*SOX9*), aggrecan (*ACN*), scleraxis (*SCX*), tenomodulin (*TNMD*), thrombospondin-4 (*THBS4*) and tenascin-C (*TNC*), were synthesized by Sango Biotech (Shanghai, China). The primer sequences are shown in Table 1. The synthesized cDNA was amplified by PCR using a LightCycler 96 system (Roche, Munich, Germany). The 2^−△△Ct^ method was used to reflect the relevant expression level of the target gene, and the expression of the target gene was normalized to that of the housekeeping gene *GAPDH*.

**TABLE 1:** Primer sequences.

Gene	5′-3′	Primer
*GAPDH*	Forward	GCAAGTTCAACGGCACAG
	Reverse	GCC​AGT​AGA​CTC​CAC​GAC​AT
*COL 1*	Forward	CGAGTATGGAAGCGAAGG
	Reverse	AGT​GAT​AGG​TGA​TGT​TCT​GG
*RUNX2*	Forward	CCC​AGT​ATG​AGA​GTA​GGT​GTC​C
	Reverse	GGG​TAA​GAC​TGG​TCA​TAG​GAC​C
*ALP*	Forward	CATCGGACCCTGCCTTAC
	Reverse	GGAGACGCCCATACCATC
*OCN*	Forward	ATT​GTG​ACG​AGC​TAG​CGG​AC
	Reverse	TCG​AGT​CCT​GGA​GAG​TAG​CC
*OPN*	Forward	GCACCACTCGCTTCTTTG
	Reverse	TTG​TTG​ATG​TCC​TGC​TCC​T
*SOX9*	Forward	GCA​CAT​CAA​GAC​GGA​GCA​A
	Reverse	GGT​TGT​AGT​GCG​GAA​GGT​TG
*ACN*	Forward	AGT​CTA​CCC​AGC​ACC​CTA​C
	Reverse	TGT​TTC​TCC​TGA​CCC​TTC​T
*SCX*	Forward	AGA​ACA​CCC​AGC​CCA​AAC​A
	Reverse	GTG​GAC​CCT​CCT​CCT​TCT​AAC
*TNMD*	Forward	GGACTTTGAGGAGGATGG
	Reverse	CGCTTGCTTGTCTGGTGC
*THBS4*	Forward	AAT​ACC​ATC​CCT​GCT​ACC​C
	Reverse	TTCCGACACTCGTCAACA
*TNC*	Forward	AAC​CAC​AAG​AAA​TAA​CCC​TC
	Reverse	TGT​TGC​TAT​GGC​ACT​GAC​T

### Western Blot

At 7 and 14 days after culture, total proteins (*n* = 3 per group) were extracted by lysing the cells in Radio-Immunoprecipitation Assay buffer (Servicebio, Wuhan, China) containing protease inhibitors (Servicebio, Wuhan, China) on ice. The supernatants were obtained by centrifugation at 12,000 rpm for 10 min, and protein concentration were quantified by the BCA assay (Servicebio, Wuhan, China). Samples containing 20 μg of protein were loaded onto 10% SDS-PAGE and then were electrotransferred to polyvinylidene difluoride membranes (Millipore, MA, United States). After blocking with 5% bovine serum albumin for 30 min, membranes were incubated with primary antibodies against *β*-actin (1:1000, gb12001, Servicebio, China), RUNX2 (1:1000, bs-1134R, Bioss, Beijing, China), ALP (1:1000, gb11528, Servicebio, Wuhan, China), SOX9 (1:1000, gb11280, Servicebio, Wuhan, China), TNMD (1:1000, ab203676, Abcam, MA, United States) and THBS4 (1:1000, ab263898, Abcam, MA, United States) overnight at 4 °C. Next, the membranes were washed and incubated with a secondary antibody (1:3000, Servicebio, Wuhan, China) for 30 min at 25°C. After washing with Tris-buffered saline containing 0.1% Tween-20, the target protein bands were detected using a chemiluminescence imaging system (ChemiScope 6,300, Clinx, Shanghai, China) after reaction with chemiluminescence substrate (ECL, Servicebio, Wuhan, China), and their intensities were measured with AlphaEaseFC software (Alpha Innotech, TX, United States) and then normalized to *β*-actin.

### Statistical Analysis

All data were expressed as mean ± standard deviation (SD). An unpaired two-tailed Student’s t-test was used to compare the two groups. One-way analysis of variance was used to compare three or more groups. A value of *p* < 0.05 was considered to be statistically significant, and the statistical analysis was performed with SPSS Statistics software (version 24, IBM, NY, United States).

## Results

### Characterization of sDCB and sDCB-ECM

**FIGURE 1:**
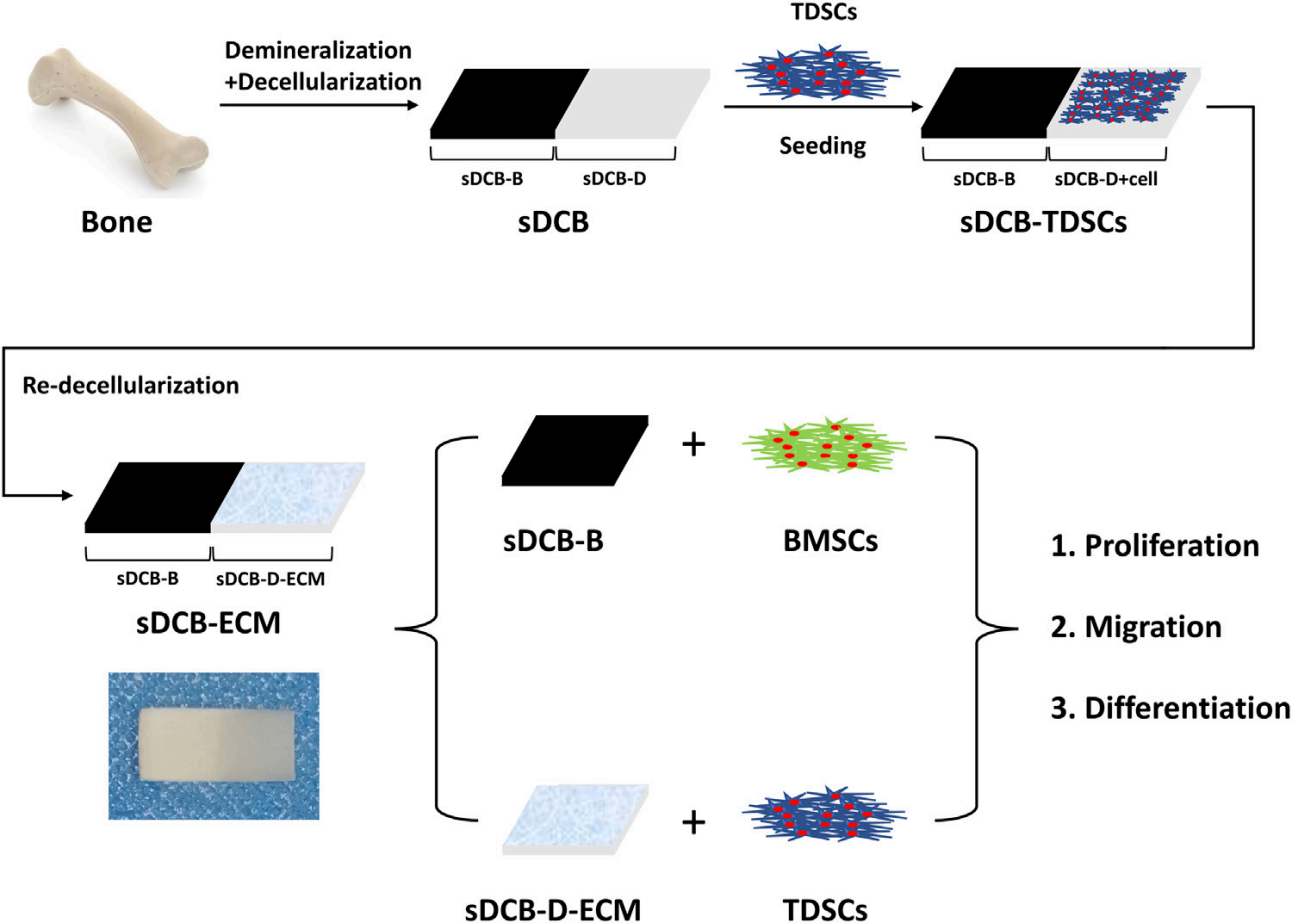
Schematic illustration of the preparation of sDCB-ECM and *in vitro* study.

**FIGURE 2:**
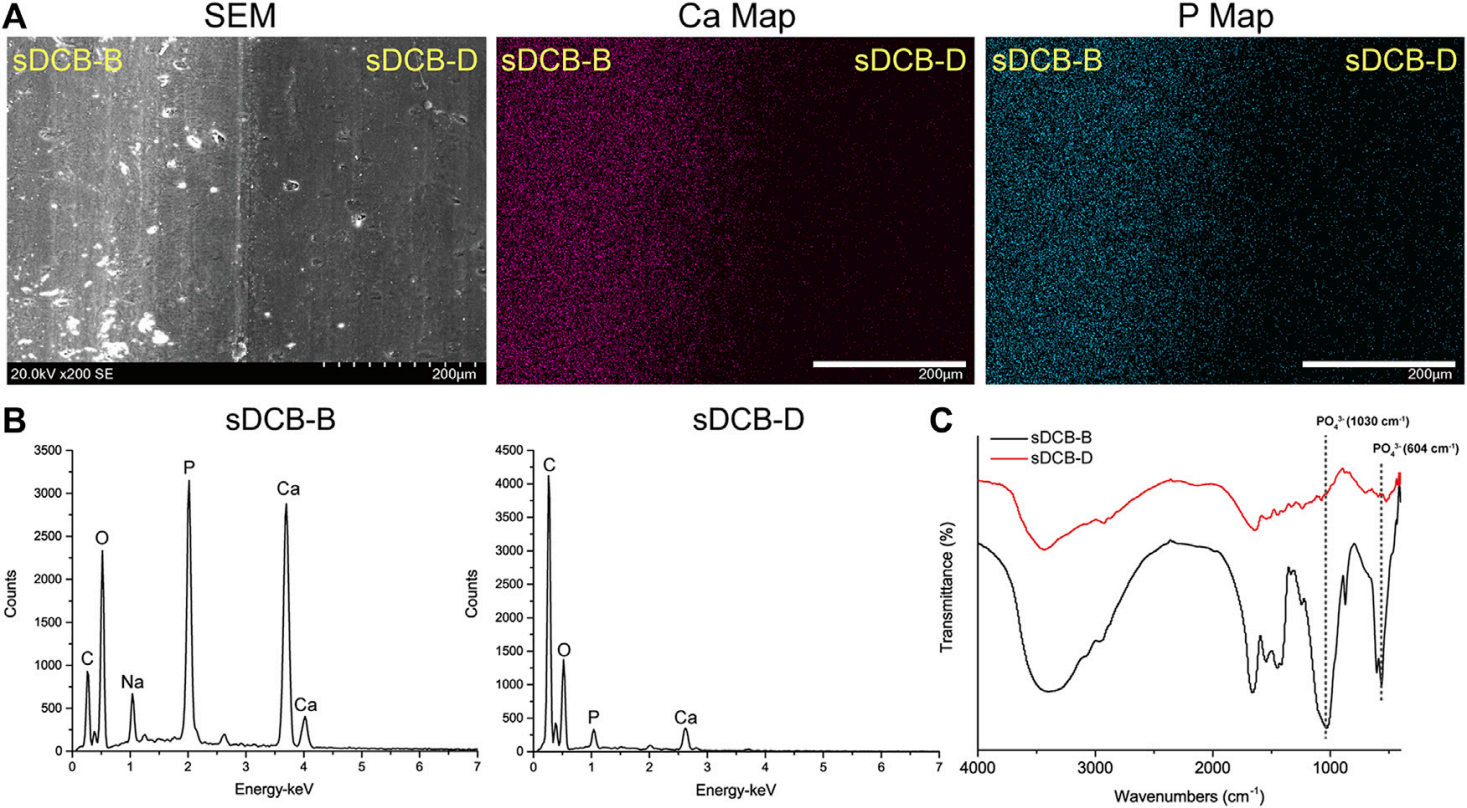
Characterization of the sDCB. **(A)** SEM image and EDX mapping of Ca and P elements in sDCB. Scale bar = 200 μm. **(B)** EDX analysis in two different regions of sDCB. **(C)** FT-IR spectra of two different regions of sDCB.

**FIGURE 3:**
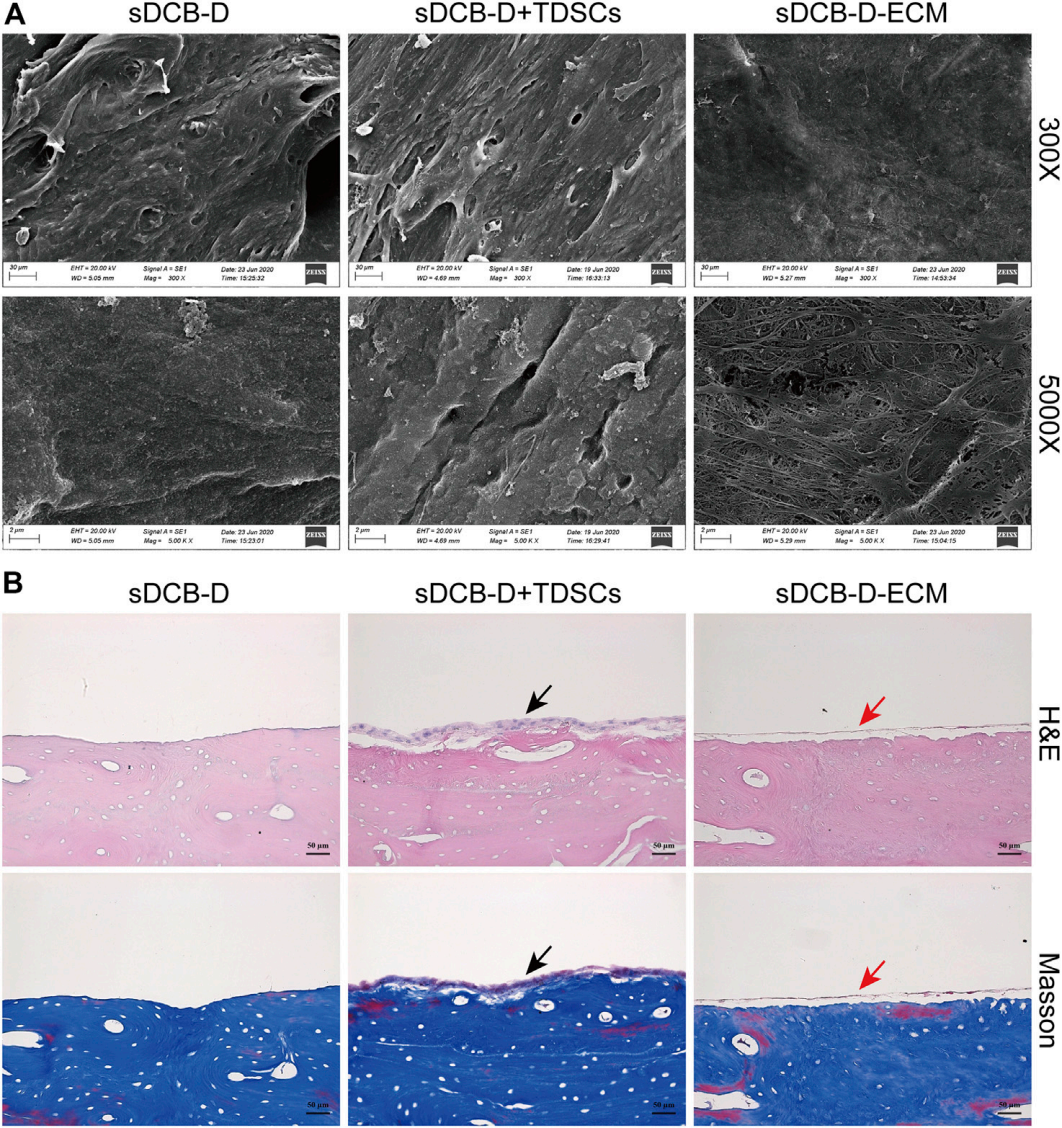
**(A)** SEM images of sDCB-D (left panels), sDCB-D with TDSCs (middle panels) and sDCB-D-ECM (right panels). Scale bar = 30 μm at ×300 magnification; Scale bar = 2 μm at ×5,000 magnification. **(B)** Histological staining of sDCB-D (left panels), sDCB-D with TDSCs (middle panels) and sDCB-D-ECM (right panels). Black arrows indicated seeded stem cells; red arrows indicated stem cell-derived ECM. Scale bar = 50 μm.

**FIGURE 4:**
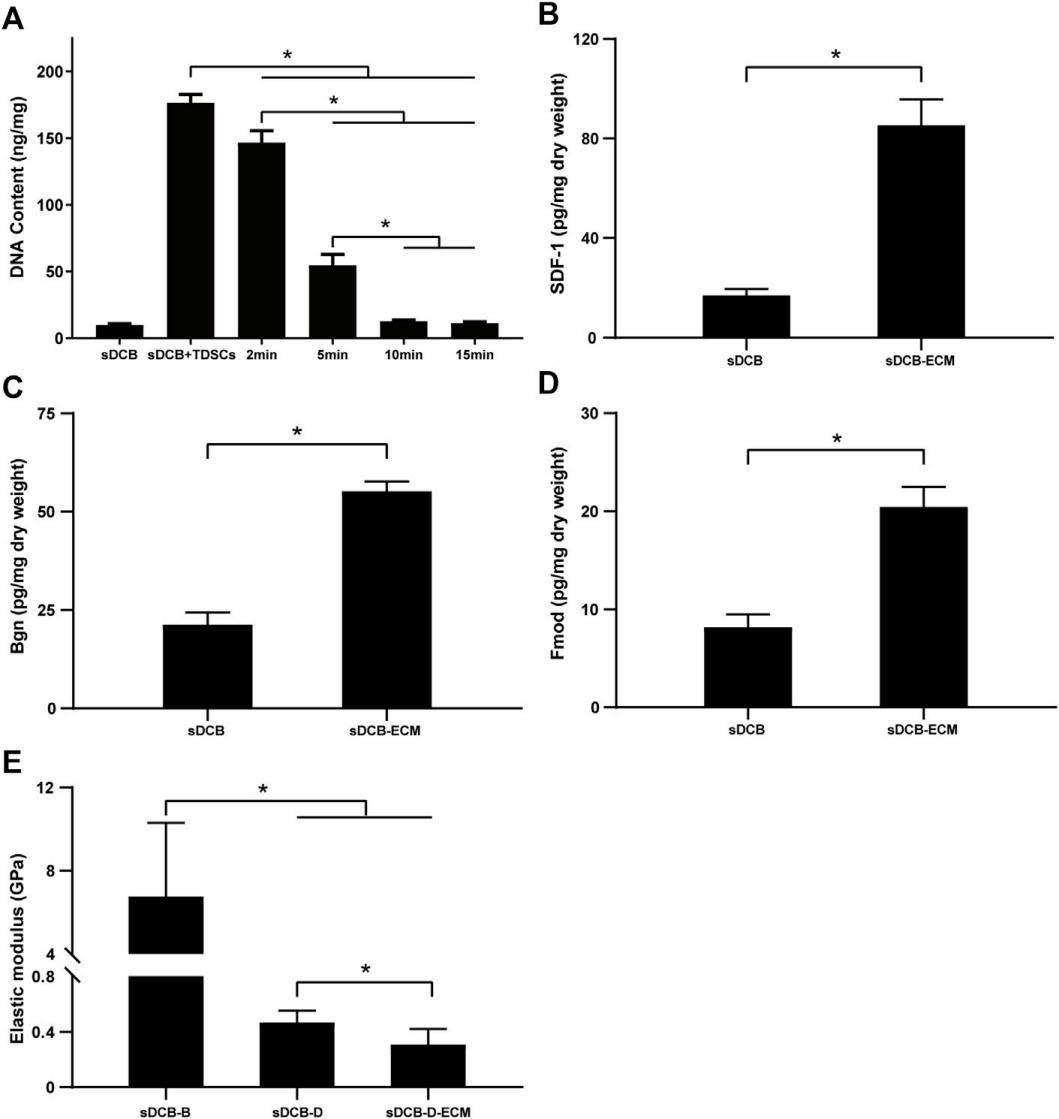
**(A)** DNA content of sDCB before and after the decellularization treatment with different time. **(B)** The contents of SDF-1 in the sDCB and sDCB-ECM. **(C)** The contents of Bgn in the sDCB and sDCB-ECM. **(D)** The contents of Fmod in the sDCB and sDCB-ECM. **(E)** The surface elastic modulus of sDCB-B, sDCB-D and sDCB-D-ECM. * Indicated *p* < 0.05.

The sDCB-ECM scaffold was prepared as illustrated in Figure 1. The SEM results showed that sDCB contained two different regions (sDCB-B and sDCB-D), and EDX elemental mapping demonstrated that there was a gradient of Ca and P elements in the sDCB (Figures 2A,B). The FT-IR results indicated that the absorptions from phosphate vibrations were not observed in the sDCB-D region. In contrast, the absorptions from phosphate vibrations were observed in the sDCB-B region (Figure 2C). Before modification, the collagen of demineralized bone was evident in sDCB-D (Figures 3A,B, left panels). After TDSCs were cultured for 2 weeks, a thin layer of cells with their ECM could be observed on the surface of the sDCB-D region (Figures 3A,B, middle panels). After decellularization, a thin layer of stem cell-derived ECM was left on the surface of the sDCB-D-ECM (Figures 3A,B, right panels). The results of SEM and histological staining showed that the stem cell-derived ECM was preserved on the surface of the sDCB-ECM after decellularization treatment (Figures 3A,B, right panels). After decellularization treatment at different time points, we found that 10 and 15 min of Triton X-100 treatment effectively removed cellular and nuclear material (*p* < 0.05, Figure 4A). For biochemical components of TDSC-derived ECM, the ELISA results indicated that SDF-1, Bgn, and Fmod contents in the sDCB-ECM were significantly higher than those in the sDCB (*p* < 0.05, Figures 4B–D). For the surface elastic modulus, the value of sDCB-B was significantly higher than that of sDCB-D and sDCB-D-ECM (*p* < 0.05, Figure 4E), and the value of sDCB-D was higher than that of sDCB-D-ECM (*p* < 0.05, Figure 4E).

### Cell Proliferation, Migration and Differentiation

**FIGURE 5:**
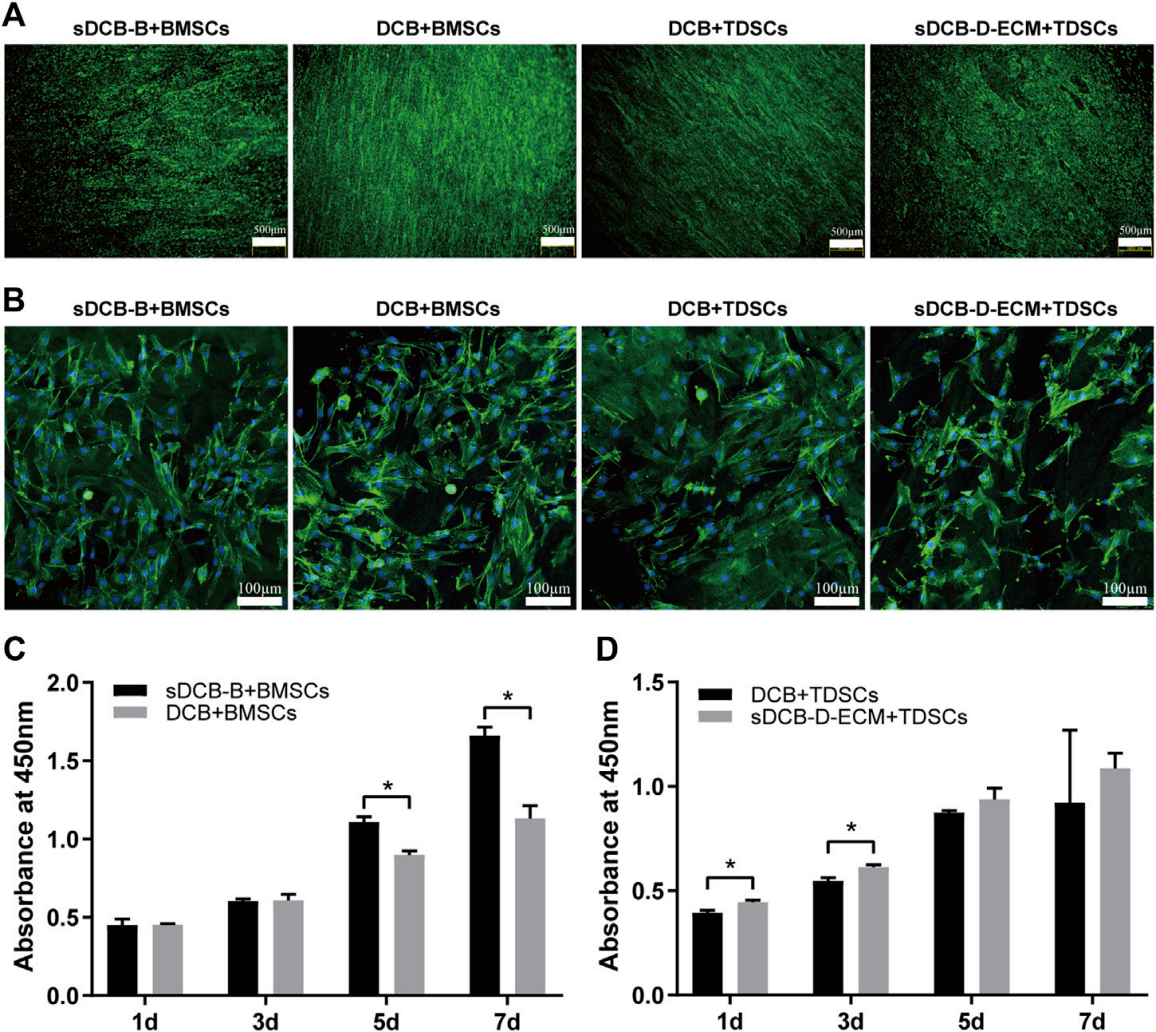
The cell morphology and viability of BMSCs and TDSCs cultured on different scaffolds. **(A)** Live/Dead cell staining of BMSCs and TDSCs cultured on sDCB-B and sDCB-D-ECM. Scale bar = 500 μm. **(B)** The cytoskeleton staining of BMSCs and TDSCs cultured on scaffolds. Scale bar = 100 μm. **(C)** The cell proliferation analysis of BMSCs cultured on sDCB-B and DCB. * Indicated *p* < 0.05. **(D)** The cell proliferation analysis of TDSCs cultured on DCB and sDCB-D-ECM. * Indicated *p* < 0.05.

**FIGURE 6:**
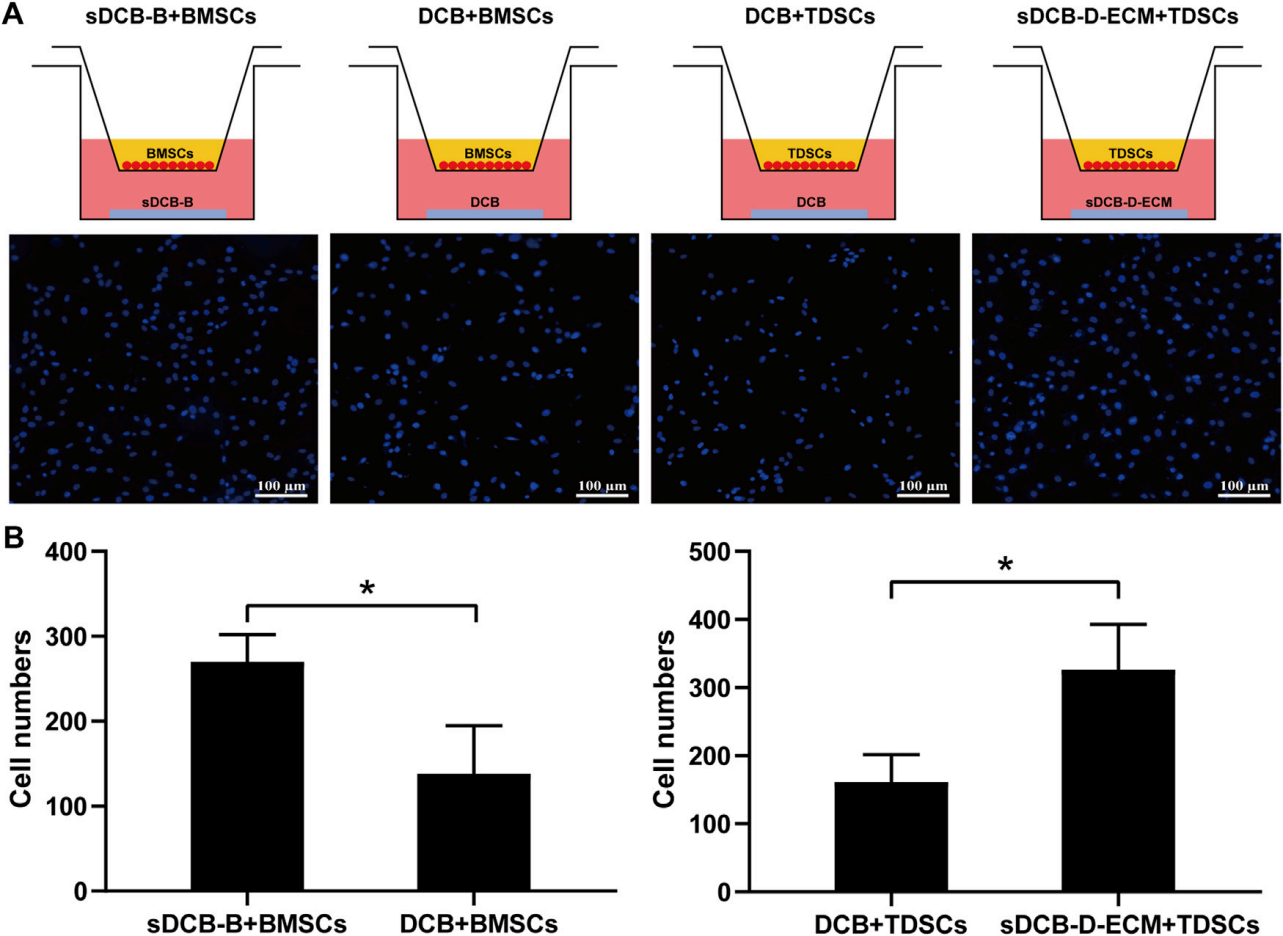
Transwell assay analysis of the effects of sDCB-B on BMSCs migration as well as sDCB-D-ECM on TDSCs migration. **(A)** Fluorescence staining of migrated BMSCs and TDSCs. Scale bar = 100 μm. **(B)** Quantitative analysis of the number of migrated stem cells between groups. * Indicated *p* < 0.05.

The results of Live/Dead cell staining confirmed that sDCB-B and sDCB-D-ECM regions of sDCB-ECM were suitable for the growth of BMSCs and TDSCs (Figure 5A). Moreover, the results of cytoskeleton staining revealed that BMSCs cultured on sDCB-B showed a polygonal shape, while TDSCs cultured on sDCB-D-ECM showed a shape of spindle (Figure 5B). After 1 and 3 days of growth, there was no significant difference in the number of BMSCs between the sDCB-B and DCB groups (Figure 5C). However, the number of cells in the sDCB-B group was significantly higher than that in the DCB group at 5 and 7 days after culture (*p* < 0.05, Figure 5C). At 1 and 3 days after culture, the number of TDSCs in the sDCB-D-ECM group was higher than that in the DCB group (*p* < 0.05, Figure 5D). However, there was no significant difference in the number of cells between the DCB and sDCB-D-ECM groups after 5 and 7 days of culture (Figure 5D). For cell migration analysis, the results showed that the number of migrated BMSCs in the sDCB-B group was significantly higher than that in the DCB group (*p* < 0.05, Figures 6A,B), and the number of migrated TDSCs in the sDCB-D-ECM group was significantly higher than that in the DCB group (*p* < 0.05, Figures 6A,B).

**FIGURE 7:**
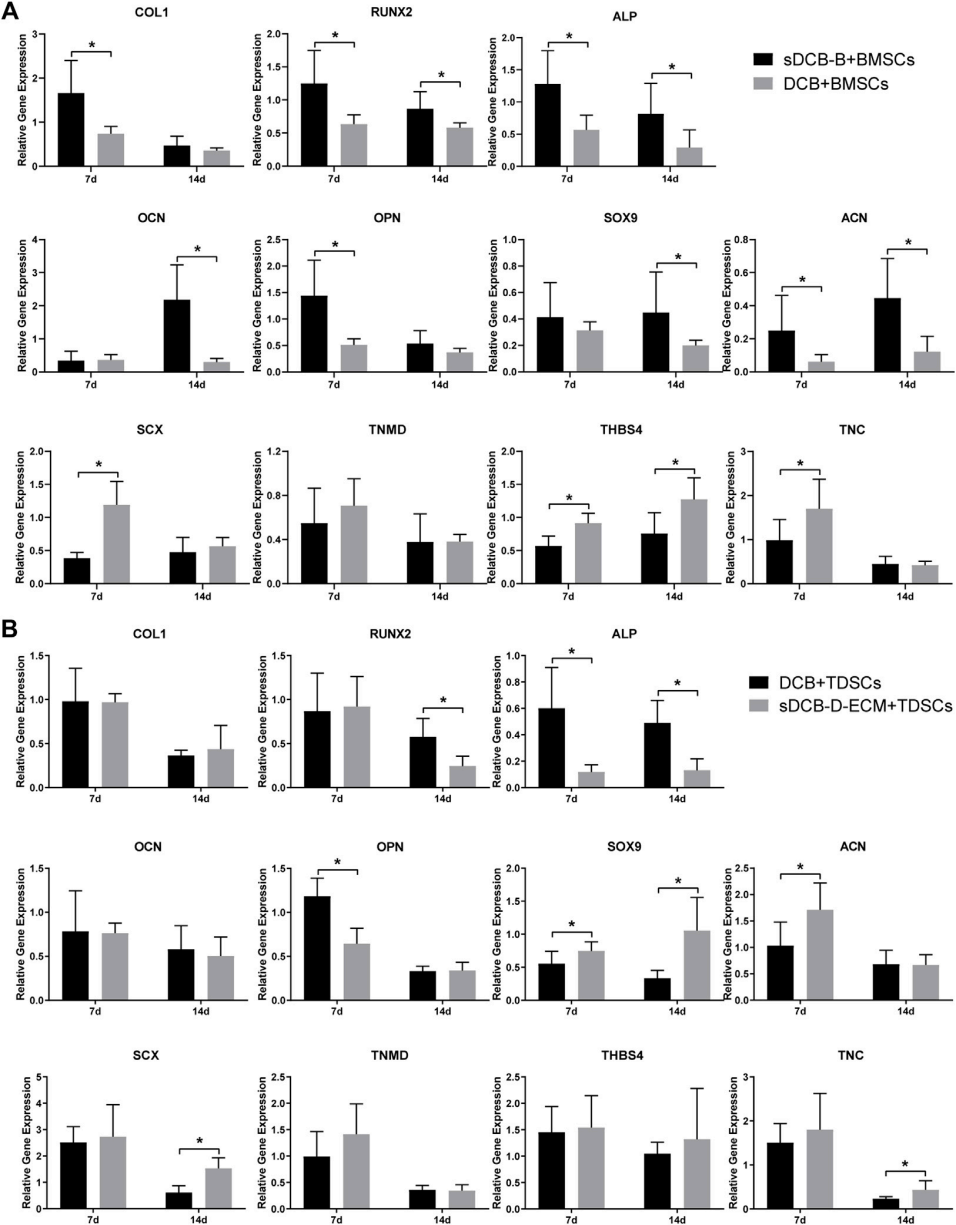
PCR analysis of relative gene expressions in BMSCs **(A)** and TDSCs **(B)** at 7 and 14 days after cultured on scaffolds. The levels of gene expression are normalized to GAPDH. * Indicated *p* < 0.05.

For differentiation-related gene expression in BMSCs, the expressions of *COL1*, *RUNX2*, *ALP*, *OPN* and *ACN* at 7 days after culture were significantly higher in the sDCB-B group than in the DCB group (*p* < 0.05, Figure 7A). However, the expressions of *SCX*, *THBS4* and *TNC* were significantly lower in the sDCB-B group than those in the DCB group (*p* < 0.05, Figure 7A). At 14 days after culture, the expressions of *RUNX2*, *ALP*, *SOX9* and *ACN* were significantly higher in the sDCB-B group than in the DCB group (*p* < 0.05, Figure 7A). However, the expression of *THBS4* was significantly lower in the sDCB-B group than in the DCB group (*p* < 0.05, Figure 7A). For differentiation-related gene expression in TDSCs, at 7 days after culture, the sDCB-D-ECM group showed higher *SOX9* and *ACN* expressions than the DCB group (*p* < 0.05, Figure 7B). In contrast, the sDCB-D-ECM group showed lower *ALP* and *OPN* expressions than the DCB group (*p* < 0.05, Figure 7B). At 14 days after culture, the sDCB-D-ECM group showed higher *SOX9*, *SCX* and *TNC* expressions than the DCB group (*p* < 0.05, Figure 7B). In contrast, the sDCB-D-ECM group showed lower expressions of *RUNX2* and *ALP* than the DCB group (*p* < 0.05, Figure 7B).

**FIGURE 8:**
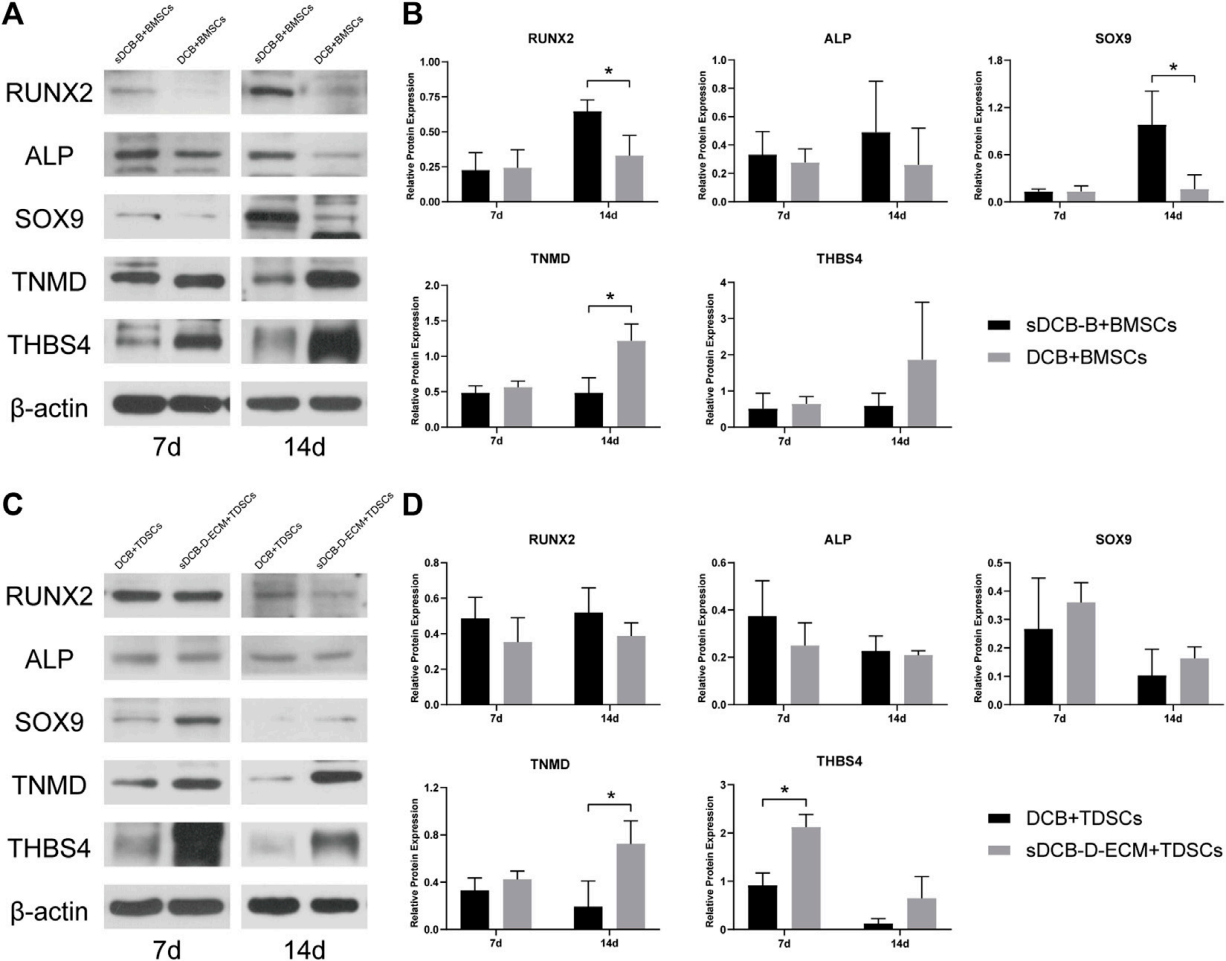
Western blot analysis of relative protein expressions in BMSCs **(A,B)** and TDSCs **(C,D)** at 7 and 14 days after cultured on scaffolds. The levels of protein expression are normalized to *β*-actin. * Indicated *p* < 0.05.

For protein expression in BMSCs, we observed that the expressions of RUNX2 and SOX9 were significantly higher in the sDCB-B group than those in the DCB group (*p* < 0.05, Figures 8A,B) at 14 days after culture, but the expression of TNMD was significantly lower in the sDCB-B group than that in the DCB group (*p* < 0.05, Figures 8A,B). However, there was no significant difference in the expressions of RUNX2, ALP, SOX9, TNMD and THBS4 between the two groups at 7 days after culture (Figures 8A,B). For protein expression in TDSCs, we found that the expression of THBS4 was significantly lower in the DCB group than that in the sDCB-D-ECM group (*p* < 0.05, Figures 8C,D) at 7 days after culture. In addition, TNMD expression was significantly lower in the DCB group than in the sDCB-D-ECM group (*p* < 0.05, Figures 8C,D) at 14 days after culture. However, there was no significant difference in the expressions of RUNX2, ALP and SOX9 between the two groups (Figures 8C,D).

## Discussion

Our study successfully prepared sDCB-ECM, which had a gradient of Ca and P elements and TDSC-derived ECM. The ELISA results showed that sDCB-ECM contained the chemokine (SDF-1) and proteoglycans (Bgn and Fmod), and the AFM results indicated that sDCB-ECM had two different surface elastic moduli. Cell viability analysis demonstrated that both sDCB-B and sDCB-D-ECM, as the two regions of sDCB-ECM, could enhance the proliferation of stem cells when compared to DCB. The results of the transwell migration assay indicated that sDCB-B and sDCB-D-ECM recruited more stem cells than DCB. PCR and western blot analyses showed that sDCB-B promoted osteogenic and chondrogenic differentiation of BMSCs, and sDCB-D-ECM promoted chondrogenic and tenogenic differentiation of TDSCs. Collectively, the sDCB-ECM had prominent advantages over DCB, including biomimetic structure with a mineral gradient and stem cell’s ECM rich in bioactive factors. The sDCB-ECM could therefore recruit more stem cells, and promoted the osteogenic and chondrogenic differentiation of BMSCs at the sDCB-B region and the chondrogenic and tenogenic differentiation of TDSCs at the sDCB-D-ECM region, which could be of a potential application for the regeneration of tendon-bone interface in the future.

It is difficult to repair the tendon-bone interface because of its hierarchical structure with a gradient of mineral content and different biochemical compositions (Boys et al., 2017). In this study, we prepared a segmentally demineralized cortical bone scaffold, characterized by a continuous collagen network from mineralized to demineralized regions. Moreover, the addition of stem cell-derived ECM changed the surface of the hierarchical cortical bone scaffold with biochemical components, such as biological chemokines and proteoglycans. Compared with DCB, this new scaffold (sDCB-ECM) had a gradient of mineral content, biological components, and surface elastic modulus, might be a promising bioscaffold to enhance the tendon-bone interface regeneration.

Previous studies found that the hydroxyapatite content of scaffolds could stimulate cell proliferation (Zambuzzi et al., 2011; Zou et al., 2012; Parisi et al., 2020). Our results also showed that sDCB-B promoted the proliferation of BMSCs at 5 and 7 days after culture when compared to DCB. In addition, a recent study showed that the morphology of BMSCs changed from being more rounded on demineralized regions to more elongated on mineralized regions (Zhou et al., 2020), similar to the findings from our study. The reason of morphological differences in BMSCs cultured on sDCB-B and DCB was probably due to changes in material stiffness (Chaudhuri et al., 2016; Mao et al., 2016). Our results indicated that the surface elastic modulus of sDCB-B was much higher than that of DCB (sDCB-D). For cell migration, our results demonstrated that sDCB-B could recruit more BMSCs than DCB, probably due to the presence of chemokines in mineralized cortical bone. For cell differentiation, our results indicated that sDCB-B promoted osteogenic and chondrogenic differentiation of BMSCs, while it inhibited the tenogenic differentiation of cells when compared to the DCB. The acceleration in osteogenic and chondrogenic differentiation of BMSCs may be due to the presence of hydroxyapatite and bone morphogenetic protein (BMP), and the presence of hydroxyapatite, BMP-2 and BMP-6 in the bone have been reported to direct osteogenic and chondrogenic differentiation of BMSCs and promote new bone and cartilage formation (Estes et al., 2006; Garrison et al., 2010; Blair et al., 2017; Rogina et al., 2017). Several studies have found a dose-dependent relationship between Ca/P elements released from scaffolds and osteochondrogenic differentiation of stem cells, and a higher dose of Ca/P promoted osteogenic differentiation but inhibited chondrogenic differentiation (Gigout et al., 2005; Wang et al., 2016). The difference between the results of our study and those of previous studies may be due to the different settings of the control group. In addition to Ca/P elements, the elastic modulus or stiffness of the scaffold also affected cell differentiation. Previous studies have found that stem cells would differentiate into muscle cells on soft substrates, and harder substrates would promote osteogenic differentiation of stem cells (Engler et al., 2006; Olivares-Navarrete et al., 2017; Zhang et al., 2018). In this study, we found that the high stiffness or elastic modulus of sDCB-B enhanced osteogenic differentiation but decreased tenogenic differentiation of BMSCs. However, the optimal elastic modulus or stiffness for stem cells to differentiate into tenocytes, chondrocytes, or osteoblasts has not been determined.

Heterotopic ossification is a problem in soft-tissue regeneration. Our previous studies demonstrated that H_2_O_2_-treated DCB had a complete collagen structure and reduced osteogenesis, which can be used for tendon repair (Yang et al., 2018a; Qing et al., 2019). However, we found that H_2_O_2_ removes some biochemical components of DCB, such as vascular endothelial growth factor and TGF-*β*1, inducing chondrogenic or tenogenic differentiation of stem cells. Stem cell-derived ECM could provide a special microenvironment to promote the healing of injured tissues (Yang et al., 2018b; Carvalho et al., 2019; Liu et al., 2020). Therefore, we used TDSC-derived ECM to modify the sDCB-D region of sDCB for improving the chondrogenic or tenogenic differentiation of stem cells.

For cell proliferation, our result showed that sDCB-D-ECM facilitated the proliferation of TDSCs at 1 and 3 days after culture when compared to DCB. The short stimulation time in cell proliferation may be due to the explosive release of biochemical factors from cell-derived ECM (Katagiri et al., 1990; Fujii et al., 2010). Our recent study also demonstrated that the chemokines (SDF-1) in ECM-modified tendon slices showed an initial burst release within 12 h (Yao et al., 2019). A previous study showed that BMSCs seeded on the demineralized region deposited thicker and more aligned collagen than those on the mineralized region (Zhou et al., 2020). In addition, TDSCs seeded on the aligned scaffold displayed an elongated shape of spindle (Ning et al., 2015). Likewise, our results also indicated that TDSCs were more elongated on sDCB-D-ECM than on DCB. For cell migration, our results indicated that sDCB-D-ECM could recruit more TDSCs than DCB, due to the presence of SDF-1 in cell-derived ECM. As is well known, SDF-1 is a crucial chemokine in cell migration, particularly in injured tissues (Wang et al., 2017). For TDSCs cultured on DCB and sDCB-D-ECM scaffolds, the results of PCR and western blot indicated that the sDCB-D-ECM promoted chondrogenic and tenogenic differentiation of cells, but inhibited osteogenic differentiation of cells when compared to DCB. The acceleration of chondrogenic and tenogenic differentiation of TDSCs may be due to the presence of Bgn and Fmod in the stem cell-derived ECM. Previous studies reported that Bgn could induce tenogenic differentiation of TDSCs at low concentrations (Song et al., 2012; Zhang et al., 2019). Moreover, Bgn and Fmod were found to maintain the function of tendon and articular cartilage, while loss of Bgn or Fmod could lead to osteoarthritis (Embree et al., 2010). In addition to biochemical factors, the elastic modulus or stiffness of scaffold also affected cell differentiation. Our study showed that stem cell-derived ECM reduced the surface elastic modulus of DCB, and the change in elastic modulus would contribute to the specific differentiation of TDSCs cultured on sDCB-D-ECM.

However, there are several limitations in our study. First, more biochemical components in stem cell-derived ECM need to be examined. Second, the pore area and swelling ratio of different regions of the scaffold may affect cell differentiation. Third, the role of stem cell-derived ECM on other materials, such as bone, needs to be investigated. Finally, in the future, there is a need to perform *in vivo* studies to verify the effect of sDCB-ECM on the tendon-bone interface repair.

In summary, we prepared sDCB-ECM composed of sDCB and TDSC-derived ECM, which has a gradient of mineral content, biological components and surface elastic modulus. Our study demonstrated that sDCB-B and sDCB-D-ECM promote the cell proliferation of BMSCs and TDSCs, and the two regions of sDCB-ECM also can recruit more stem cells than DCB. In addition, sDCB-B can stimulate osteogenic and chondrogenic differentiation of BMSCs, and sDCB-D-ECM can stimulate chondrogenic and tenogenic differentiation of TDSCs when compared to DCB. Our study shows that sDCB-ECM may be a potential bioscaffold to enhance the tendon-bone interface regeneration.

## Data Availability

The original contributions presented in the study are included in the article/Supplementary Material, further inquiries can be directed to the corresponding author.
